# BMSCs Interactions with Adventitial Fibroblasts Display Smooth Muscle Cell Lineage Potential in Differentiation and Migration That Contributes to Neointimal Formation

**DOI:** 10.1155/2016/3196071

**Published:** 2016-01-06

**Authors:** Y. Wendan, J. Changzhu, S. Xuhong, C. Hongjing, S. Hong, Y. Dongxia, X. Fang

**Affiliations:** ^1^College of Basic Medicine, Binzhou Medical University, Yantai, Shandong 264003, China; ^2^Emergency Department of Cardiology, The Second People's Hospital of Jining, Shandong 272000, China

## Abstract

In this study a model of simulated vascular injury in vitro was used to study the characterization of bone-marrow-derived mesenchymal stem cells (BMSCs) morphology and to investigate the differentiation and migration of BMSCs in the presence of adventitial fibroblasts. BMSCs from rats were indirectly cocultured with adventitial fibroblasts in a transwell chamber apparatus for 7 days, and clonogenic assays demonstrated that BMSCs could be differentiated into smooth muscle-like cells with this process, including smooth muscle *α*-actin (*α*-SMA) expression by immunofluorescence staining. Cell morphology of BMSCs was assessed by inverted microscope, while cell proliferation was assessed by MTT assay. The expressions of TGF-*β*1, MMP-1, and NF-*κ*B were detected by immunofluorescence staining and Smad3 mRNA was measured by reverse transcription PCR. Migration ability of BMSCs with DAPI-labeled nuclei was measured by laser confocal microscopy. Our results demonstrate that indirect interactions with adventitial fibroblasts can induce proliferation, differentiation, and migration of BMSCs that can actively participate in neointimal formation. Our results indicate that the pathogenesis of vascular remodeling might perform via TGF-*β*1/Smad3 signal transduction pathways.

## 1. Introduction

Vascular injury and vascular restenosis, as well as angiogenesis, are the leading causes of coronary arteries and cardiovascular diseases. Vascular remodeling plays an important role in the pathology in this process. The prevailing belief is that hyperplasia of the vascular intima and tunica media contributes to the formation of intimal vascular restenosis or atherosclerotic lesion, while the role of vascular adventitia has long been neglected. Recent evidences have demonstrated that vascular adventitia, specifically the fibroblasts, may serve as a critical regulator of postinjury vascular restenosis and atherosclerotic lesion [1, 2]. Moreover, results from experiments within our laboratory [3, 4] suggested that proliferative changes of adventitial fibroblasts and synthesis of cytokines in the early stages of atherosclerotic lesion have been detected. In this hyperplasia process, an activation of adventitial fibroblasts into myofibroblasts tends to migrate into the intima. Additional factors associated with this process including synthesis transforming growth factor-beta 1 (TGF-*β*1) and collagen [5, 6].

However, it has also been reported that some BMSCs participate in the adhesion and differentiation processes of vascular restenosis [7]. More recently, accumulating findings have indicated that BMSCs could enter the circulation at the site of vascular injury to participate in the development of hyperplasia [8, 9]. Restenosis is a major complication of coronary angioplasty and atherosclerosis of cardiovascular diseases, and possibly part of the confusion surrounding the origin and identity of contributing cell types is not well understood. In addition, adventitial fibroblasts are activated during the early stages of injury or stress and secrete large numbers of cytokines, chemokines, and enzymes [10, 11]. Some of these factors may in turn play a role in stem cell mobilization. However, the surrounding microenvironment of BMSCs differentiation is complex and is influenced by many factors including the release of autocrine and paracrine factors.

In this current experiment, we added TGF-*β*1 to the cell culture medium in order to activate the adventitial fibroblasts differentiation into myofibroblasts, to simulate the microenvironment of vascular injury in vitro, as well as to assess the effect of BMSCs indirect contact with myofibroblasts on transdifferentiation and migration. With this model, it is possible to explore the mechanisms of BMSCs involvement in the formation of vascular restenosis. In addition, these findings will provide the foundation for future work that can be applied for the study of pathogenesis of vascular restenosis and atherosclerosis after vascular injury. This study will serve as the basis for new strategies in the treatment of cardiovascular diseases.

## 2. Materials and Methods

### 2.1. Animals and Reagents

Male Sprague-Dawley (SD) rats, 6 weeks old (200–230 g), were provided by the experimental animal center of Binzhou Medical University. The experimental animals were treated according to the guidelines approved by university committee on the use and care of laboratory animals. Trizol and the two-step PCR detection kits were purchased from the TaKaRa Co. (Dalian, China). Anti-*α*-SMA antibodies, anti-TGF-*β*1 antibody, anti-MMP-1 antibody, and anti-NF-*κ*B p65 antibody for immunocytochemistry were purchased from the Boster Co. (Wuhan, China). The light microscope and camera were purchased from Nikon Corporation (Japan). All other chemicals and reagents were of analytical grade.

### 2.2. Isolation, Cultivation, and Identification of Vascular Adventitial Fibroblast

Sprague-Dawley rats were used in these experiments. Under aseptic conditions, the adventitia of thoracic aortas of the rats was carefully isolated from the media and intima. The adventitia was then cut into tissue pieces and plated on a culture dish containing complete medium (containing 15% fetal bovine serum) in a humidified atmosphere of 5% CO_2_ at 37°C. The medium was exchanged every other day until the tissue pieces were surrounded by adherent fibroblasts. The tissue pieces were then removed and cultured for two more days. When the cells reached 80% confluence they were harvested with 0.25% trypsin. Vascular adventitial fibroblasts were identified by immunohistochemical staining using the primary antibodies Vimentin, Desmin, and *α*-SMA (1 : 100). The 2-3 generations of adventitia fibroblasts were harvested and cultured in 10% fetal bovine serum on 25 mm cover slips in 6-well plates and then replated at a density of 1 × 10^4^ cells/cm^2^. An in vitro model of the vascular injury was simulated in this study by adding TGF-*β*1 to the cell medium to active adventitial fibroblasts, the model groups were inoculated with TGF-*β*1 on the cell medium to a final concentration of 10 ng/mL [12], and the medium was replaced every other day with a medium containing newly added TGF-*β*1. The adventitial fibroblasts were cultured for 1, 3, or 7 days as the model groups, respectively, while the adventitial fibroblasts were cultured without TGF-*β*1 in cell medium for 7 days served as the control group. The adventitial fibroblasts of the four groups were washed 3 times in PBS and fixed with 4% paraformaldehyde subjected to immunofluorescence staining. The cells were incubated with primary antibodies overnight at 4°C, with IgGCy3 as the second antibody in a 37°C incubator for 30 min. Reverse transcription PCR was used to determine the expressions of *α*-SMA, TGF-*β*1, MMP-1, and NF-*κ*B p65 mRNA in the adventitial fibroblasts. Total RNA was prepared with absolutely RT-PCR Kit of the TaKaRa Company. PCR primers were synthesized by the TaKaRa Company (Table 1). PCR amplification system in a total of 12.5 *μ*L was applied to the following PCR program: 5 min at 95°C (before denaturation), 30 s at 95°C (initial denaturation), 30 s at 58°C, 60 s at 72°C, repeated 30 times, and 5 min at 72°C (amplification). The RT-PCR products were assessed on 1.5% agarose gels. Results of the electrophoresis were scanned by a Gel imaging system for quantitative analysis and photographed.

### 2.3. Isolation, Cultivation, and Identification of BMSCs

Under aseptic conditions, bilateral femurs of rats were removed and isolated. Bone marrow was rinsed with Dulbecco's Minimum Essential Medium (DMEM) and centrifuged at 1,000 rpm for 10 min. The supernatant was discarded and the precipitate resuspended with PBS solution; 6 mL of this solution was gradually applied onto 6 mL of lymphocyte separation medium and centrifuged at 1,000 rpm for 10 min. The cells in the resultant middle white layer were carefully extracted, washed with PBS, and plated on a culture dish in a humidified atmosphere of 5% CO_2_ in air at 37°C in complete medium (containing 15% fetal bovine serum). The culture medium was exchanged every two days. The growth and morphology of cells were assessed with an inverted microscope. After reaching confluence, the cells were subcultured and the 2-3 generations of cells were used for FACS analysis. The cells were harvested by incubation with trypsin and centrifuged briefly to obtain a cell pellet. The harvested cells were then washed with ice-cold PBS once and centrifuged for 5 minutes at 1500 rpm and room temperature. The cell pellet was then resuspended with 100 *μ*L ice-cold PBS. Cells were labelled with antibodies against CD106, CD44, CD45, and CD34 and analysed with a FACSscan flow cytometer.

### 2.4. BMSCs Indirect Coculture with Vascular Adventitial Fibroblasts

The 2-3 generations of adventitial fibroblasts were seeded in transwell inserts with 0.4 *μ*m pore membrane filters and allowed to attach onto the surface, the cells were also cultured in the presence of 10 ng/mL TGF-*β*1 in 15% FBS medium (a model of simulated vascular injury in vitro was used by activation of TGF-*β*1 to adventitial fibroblasts for 3 days) to induce differentiation into myofibroblasts, and after 3 days the cell medium was then replaced with new medium containing 10% FBS without TGF-*β*1, while BMSCs were seeded and subsequently loaded onto the upper chamber at 2 × 10^4^ cells/mL. BMSCs were indirectly cocultured with fibroblasts for 1, 3, or 7 days as the model groups, respectively, while BMSCs were cultured alone for 7 days that served as controls; the upper side of the filters was carefully washed with PBS, and cells on the underside of the membrane were fixed with 4% paraformaldehyde for 10 mins. BMSCs were labeled with the nuclear dye DAPI (0.02 g/L fluorescent dye 4,6-acetyl 2-2-phenyl indole, Santa Cruz Biotech) overnight and then were identified by immunohistochemical staining using the primary antibodies *α*-SMA (1 : 200), TGF-*β*1 (1 : 500), or anti-rabbit IgG-Smad2/3 with IgGCy3 (1 : 200) as the fluorescence labeling goat and subjected to gene expression analysis by using RT-PCR. PCR primers were shown in Table 1.

### 2.5. Proliferation of BMSCs

BMSCs proliferation activity was evaluated using the MTT (3-(4,5-dimethylthiazol-2-yl)-2,5-diph-enyl-2H-tetrazolium bromide) assay. BMSCs were divided into the model groups in which cells were indirectly cocultured for 1, 3, or 7 days, respectively, while BMSCs were cultured alone for 7 days that served as controls. BMSCs were then harvested during the logarithmic growth phase and seeded in 96-well plates at a density of 1 × 10^4^ cells/cm^2^; 20 *μ*L of MTT solution (5 mg/mL) was added to each well and the cells were then incubated for an additional 4 h. The culture supernatant was removed and 150 *μ*L of dimethyl sulfoxide was added to each well to fully dissolve the MTT-formazan crystals. Cell proliferation was determined by measuring the absorbance (Abs) at *λ* = 490 nm using a microplate reader.

### 2.6. Migration of BMSCs

Migration ability of BMSCs was measured in a transwell chamber apparatus with 8 *μ*m pore membrane (Costar, New York, USA). Briefly, the cell suspension (1 × 10^4^ cells/cm^2^) of adventitial fibroblasts was loaded into the lower compartment of the chamber with medium containing 10% FBS with TGF-*β*1; after differentiation into myofibroblasts for 3 days, the cell medium was then replaced with a new medium without TGF-*β*1, whereas BMSCs were seeded in the upper compartment of the chamber. The filters were maintained at 37°C in a humidified air atmosphere containing 5% CO_2_ for 1, 3, or 7 days, while BMSCs were cultured alone for 7 days that served as controls; the filters were removed and cells remaining on the upper surface of the membrane were removed with a cotton swab. The membranes were washed with PBS two times, and cells adhering beneath the membrane were fixed in 4% paraformaldehyde and stained with DAPI. Migration ability of BMSCs was quantified by cell counts of five random fields at 100 magnifications in each membrane. Laser confocal microscopy was used for fluorescent analysis. The number of positive stained cells of BMSCs was counted and expressed as the percentage of total nuclei.

### 2.7. Statistical Analysis

All experiments were conducted 3–6 times and significant differences were calculated as *P* < 0.05 compared to control by *t*-test or one-way ANOVA using SPSS software.

## 3. Results

### 3.1. Adventitial Fibroblasts Cultivation and Differentiation into Myofibroblasts

**Figure 1:**
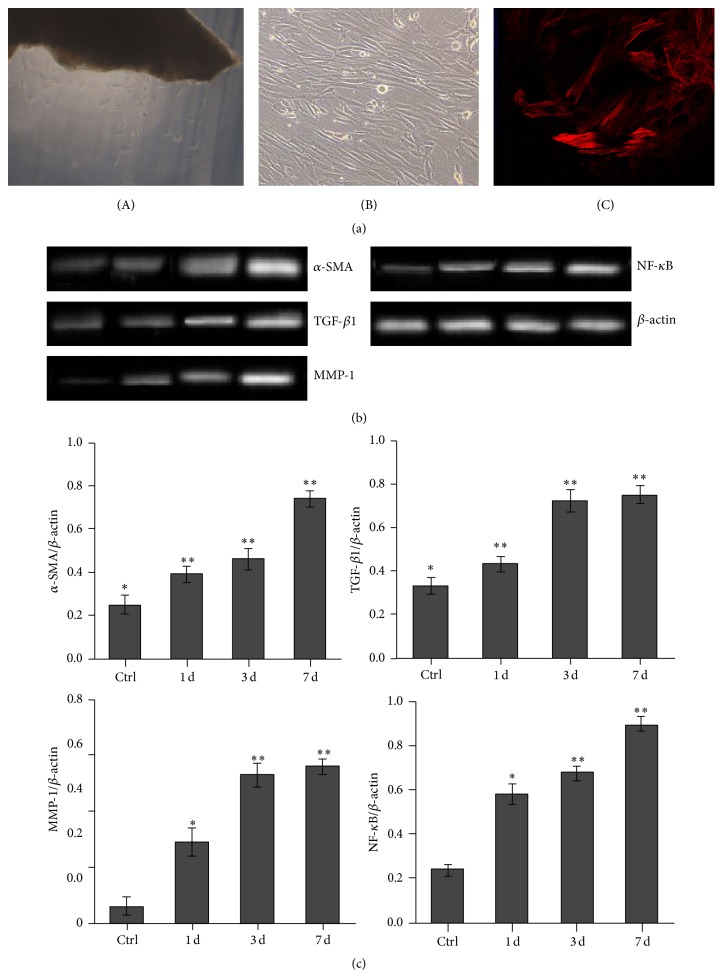
Characteristics of cultured vascular adventitial fibroblasts and expression of *α*-SMA, TGF-*β*1, MMP-1, and NF-*κ*B. (a) Morphological observation and representative photographs of fibroblasts. (A) Fibroblasts grown from tissue pieces of vascular adventitia (×100). (B) The 2-3 generations of fibroblasts showed a robust growth with a multilayer overlapping crest shape (×100). (C) Fibroblasts stained with Cy3 antibody (red) indicating intracellular *α*-SMA after TGF-*β*1 treatment (×400). (b) Expressions of *α*-SMA, TGF-*β*1, MMP-1, and NF-*κ*B mRNA in adventitial fibroblasts in the model groups or the control group with or without TGF-*β*1. (c) Significant differences among groups were indicated by RT-PCR. Averages of at least three independent experiments (*n* = 3). Bars indicated the mean ± SD. ^*∗*^ *P* < 0.05 compared to the control (model versus control, ^*∗*^ *P* < 0.05; the model group for 3 or 7 days versus 1 day, ^*∗∗*^ *P* < 0.05).

After 2-3 days of primary culture, fibroblasts were transformed from the edges of adventitial tissue pieces. Primary cultured adventitial fibroblasts exhibited adherent growth with an irregular polygonal shape. The 2-3 generations of fibroblasts rapidly proliferated and showed a robust growth with a multilayer overlapping crest shape. Staining of vascular adventitial fibroblasts with anti-*α*-SMA was negative, while anti-Vimentin and Desmin were both positive. Additionally, the *α*-SMA expression, which represents a characteristic marker of myofibroblasts, was detected in the model groups after TGF-*β*1 treatment by immunofluorescence staining (Figure 1(a)), and *α*-SMA, TGF-*β*1, MMP-1, and NF-*κ*B mRNA expressions were identified using RT-PCR. The number of these positive cells increased as a function of culture duration (Figures 1(b) and 1(c)).

### 3.2. BMSCs Morphology and Immunophenotyping

**Figure 2:**
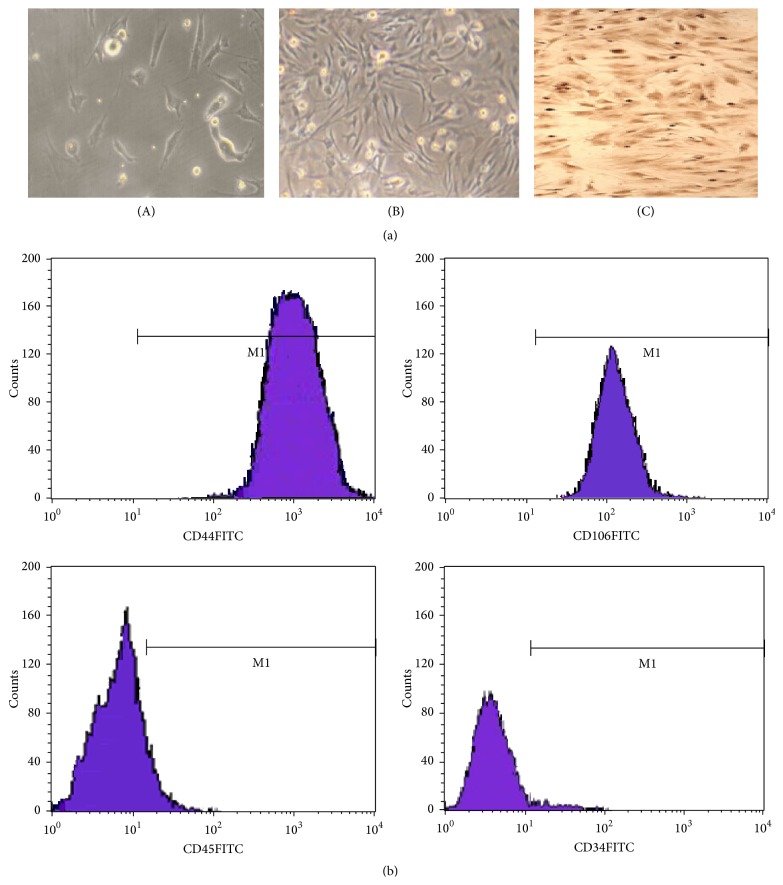
Characteristics of cultured BMSCs of morphology and immunophenotyping. (a) Morphology and immunocytochemistry staining of BMSCs. (A) The primary cultured BMSCs showed adherence to the culture dish displaying short bar shapes. (B) The 2-3 generations of BMSCs transformed into long spindles with vortex shape. (C) Immunocytochemistry expression of CD44 in the 3rd generation of BMSCs (×100). (b) Flow cytometry analysis of immunophenotype of BMSCs.

The primary cultured BMSCs were found adherent to the culture dish initially displaying short bar shapes, as observed under inverted microscope. Subsequently, BMSCs extended into a polygonal shape with diverse morphological characteristics until, eventually, their shape gradually transformed into long spindles with vortex shape (Figure 2(a)). After obtaining the ~2-3 generations of BMSCs, flow cytometry analysis was adopted and showed that BMSCs were negative for the cell surface marker CD45 and CD34, while BMSCs were positive for the mesenchymal stem cell markers CD44 and CD106 (Figure 2(b)). These results suggested that the cultured cells have similar morphological and immunophenotypical characteristics of BMSCs.

### 3.3. Myogenic Induction of BMSCs

**Figure 3:**
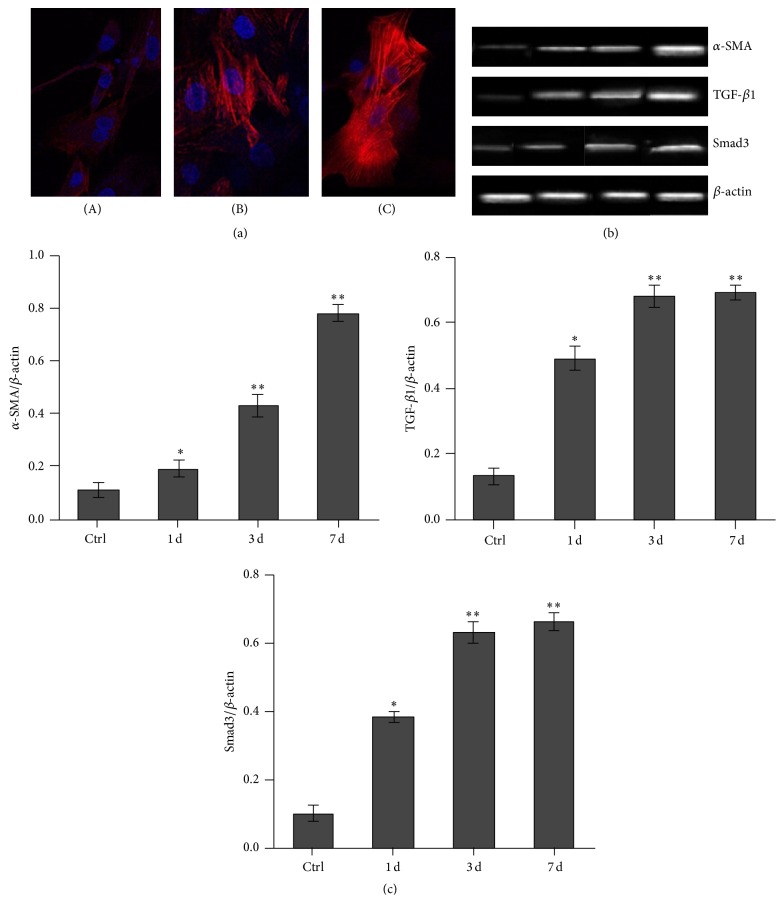
Myogenic induction of BMSCs. (a) Immunofluorescence expressions of BMSCs. (A–C) BMSC treated with anti-*α*-SMA showing a blue-stained nucleus and a red-stained cytoplasm after coculture for 1, 3, or 7 days (×400); *α*-SMA expressions of BMSCs positive rates were 23% at 1 day, 49% at 3 days, or 82% at 7 days. (b) Expressions of *α*-SMA, TGF-*β*1, and Smad3 mRNA in BMSCs showing significant differences among the four groups were indicated by RT-PCR. (c) Results represent the average of at least six independent experiments (*n* = 6). Bars indicate the mean ± SD. ^*∗*^ *P* < 0.05 compared to the control (model versus control, ^*∗*^ *P* < 0.05; the model group for 3 days or 7 days versus 1 day, ^*∗∗*^ *P* < 0.05).

The 2-3 generations of vascular adventitial fibroblasts were loaded onto the under chamber in the presence of 10 ng/mL TGF-*β*1 in 10% FBS medium. After differentiation into myofibroblasts for a period of 3 days, the culture medium was then exchanged without TGF-*β*1. The ~2-3 generations of BMSCs were loaded onto the upper chamber, the cells were indirectly cocultured with adventitial fibroblasts for 1, 3, or 7 days, and myogenic induction of BMSCs was assessed by immunofluorescence staining and PCR; they were labeled with the nuclear dye DAPI. It was observed that *α*-SMA expression, which exhibited an increasing *α*-SMA expression, gradually extended to the surrounding of cell cytoplasm and showed pseudopodia shape (Figure 3(a)). An increased expression of TGF-1 and Smad3 mRNA in BMSCs was observed in response to induction with adventitial fibroblasts, while the expressions in the control group served as controls (Figures 3(b) and 3(c)).

### 3.4. Migration of BMSCs

**Figure 4:**
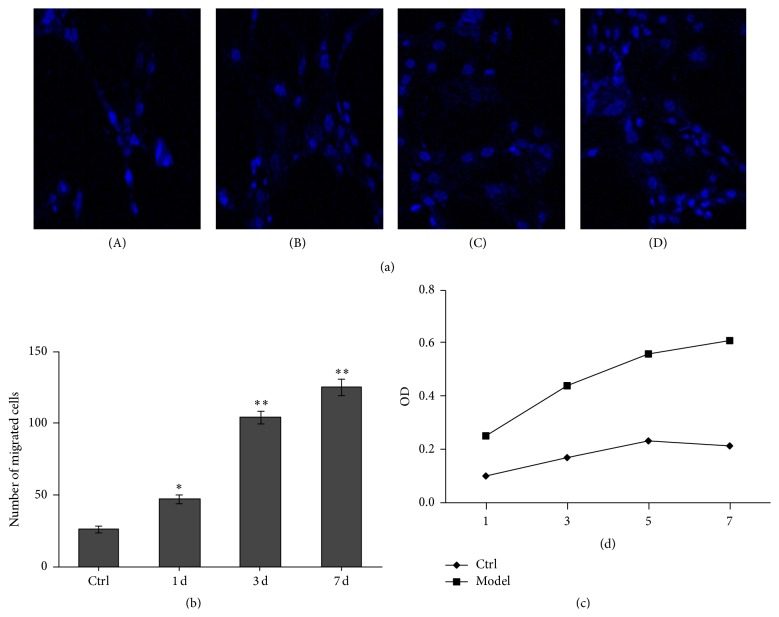
Migration and proliferation of BMSCs. (a) Migration of BMSCs. (A) BMSCs cultured alone for 7 days served as controls (×200). (B–D) Immunofluorescence expressions of BMSCs exhibited a blue-stained nucleus after coculture for 1, 3, or 7 days. (b) The number of migrated cells in every group. ^*∗*^ *P* < 0.05 compared to the control (model versus control, ^*∗*^ *P* < 0.05; the model group for 3 days or 7 days versus 1 day, ^*∗∗*^ *P* < 0.05). (c) Proliferation rates curve of BMSCs in various groups.

This assay involved a two-compartment system where cells may be induced to migrate from an upper compartment through the membrane into a lower compartment. Migration assay showed that adventitial fibroblasts on the under chamber in transwell inserts derived BMSCs on the upper chamber migrated through 8 *μ*m diameter pores during a chamber migration assay. Four groups of treatment as above, migration assays were terminated through retrieving the filter, rubbing of nonmigrated cells from top surface, and counting cells that were found on the underside of the filter. The cells were then labeled with the nuclear dye DAPI and visualized at 400x magnification (Figure 4(a)). Numbers of migrated cells in coculture groups at 7 days were significantly higher than other groups (*P* < 0.01, Figure 4(b)).

### 3.5. BMSCs Proliferation Ability

BMSCs proliferation activity in vitro was evaluated using the MTT assay. As shown in Figure 4(c), the four groups, BMSCs proliferative activity increasing as a function of exposure time was observed after coculture. Maximal proliferation rate of the respective model group was observed after coculture for 7 days (*P* < 0.05).

## 4. Discussion

Vascular restenosis represents a major component of cardiovascular diseases, mainly due to its association with hypertension and atherosclerosis, and major complication of coronary angioplasty is vascular remodeling, but the origin and identity of contributing cell types and factors involved in this process are not well understood. Adventitial fibroblasts are the main cell type in the adventitia and are activated in the early phase of vascular injury where they can change their phenotype to myofibroblasts. These myofibroblasts show characteristics of smooth muscle cells with actin in plasma and migrate toward the lumen neointimal, where they are involved in the proliferation of vascular remodeling [2]. However, recent evidences from stem cell research and atherosclerosis mechanism indicate that vascular stem/progenitor cells may be the source of smooth muscle cells that accumulate in vascular restenosis lesions, but the origin of these stem/progenitor cells is unknown. Sata et al. [9] reported that bone-marrow-derived stem cells express *α*-SMA, and these cells are involved in the formation of neointimal lesions after vascular injury. Findings from Abedi's laboratory indicated that stem cells existed in normal arterial walls and capillaries and these stem cells that are involved with circulating progenitor cells entered the circulation and perfused other tissues [12]. Hoshino et al. [13] reported that stem cells existed in vascular adventitia, and cultured vascular adventitia had characteristics of stem cells. Xu found that progenitor cells existed in blood and vascular tissue, and these cells were involved in the formation of atherosclerosis [14, 15].

Recently, the relationship between stem/progenitor cells and cardiovascular diseases has received a considerable amount of attention. BMSCs have the potential for multiple differentiations [16]. It has been reported that BMSCs have the capacity to differentiate into vascular endothelial and smooth muscle cells in vitro and in vivo [17, 18]. Special attention has been paid to BMSCs interactions with adventitial fibroblasts in transdifferentiation for vascular remodeling within our analysis [19]. With vascular injury, some cytokines and chemokines can mobilize these stem/progenitor cells into the local for adhesion of the injury site [20–22]. TGF-*β*1 represents one of the most important cytokines in this regard, as it appears to promote vascular proliferation, migration, and increasing collagen and elastin during adventitia remodeling [5, 23]. Other factors include the expressions of MMPs and NF-*κ*B, all of which contribute to regulating vascular proliferation and migration responses [24]. MMP-1 is an extracellular matrix protein hydrolysis enzyme, which participates in regulating vascular remodeling and cell migration. Activation of the nuclear transcription factor NF-*κ*B can affect a variety of gene expressions and regulations. Many promoter regions of genes encode cytokines containing transcription factors for NF-*κ*B binding sites, such as TGF-*β*1 and MMPs. As a result, NF-*κ*B activation can lead to an increase in the expression of a number of cytokines. Zhang et al. [25] reported that TGF-*β*1 induced MMP-9 expression in rat vascular smooth muscle cells via the NF-*κ*B pathway. This MMP-9 results in myofibroblast migration, through the degradation of extracellular matrix. At present, results from several studies have indicated that various signaling pathways and signal transductions are involved in driving this differentiation process, although the mechanisms through which stem cells differentiate into smooth muscle remain unclear. Smad2 or Smad3 is the positive regulatory factor of cardiovascular remodeling. Smad2 and Smad3 signaling pathways can enhance adventitial fibroblasts functions activated by TGF-*β*1 [26], and Smad3 signal transducers can act as enhancers of fibroblast collagen expression, which then increases the vascular fibrosis further [27].

In our study, the microenvironment of BMSCs indirect contact with vascular adventitial fibroblasts was simulated in vitro. The goal of these indirect cocultures enabled us to induce cell interactions among cytokines, chemokines, and enzymes, and, in this way, we observe their differentiation, migration, and phenotypic transformations. Immunofluorescence staining and RT-PCR were then performed after 1, 3, and 7 days in coculture where a strong positive expression of *α*-SMA and a positive expression of TGF-*β*1 in BMSCs were observed. Furthermore, Smad3 mRNA was significantly upregulated in BMSCs over this 7-day period. These experimental evidences suggest that when BMSCs are in indirect contact with vascular adventitial fibroblasts in vascular injury, they differentiate and migrate and promote the transformations of smooth muscle-like properties in the vascular remodeling at least partly via TGF-*β*1/Smad3 signal transduction pathways.

However, previous studies reported that BMSCs accelerate endothelial repair and inhibit vascular smooth muscle hyperplasia [28], so it remains critical to identify means of promoting beneficial angiogenesis while inhibiting smooth muscle hyperplasia and to elucidate the mechanisms of vascular remodeling. An understanding of this relationship between adventitial hyperplasia and BMSCs differentiation is of considerable importance because it will serve as a foundation for new protocols to study vascular restenosis and further identify BMSCs role in cardiovascular disease.

## 5. Conclusion

Based upon our results, it can be postulated that large amounts of bioactive substances are released in vessel injury, including cytokines or enzyme, which may not only activate fibroblast proliferation and secretion, but also induce BMSCs differentiation and migration. In this way, BMSCs and fibroblast may both participate in vascular remodeling through the processes involving differentiation to vascular smooth muscle-like cells as a result of their interactions. Such information will provide critical new targets to prevent and treat the postinjury vascular restenosis of coronary arteries and various cardiovascular diseases.

## Figures and Tables

**Table 1:** PCR primers used in this study.

Gene name	Sequence
*α*-SMA	Forward: 5′-CGAGAAGCTGCTCCAGCTATGTG-3′
	Reverse: 5′-CTCTCTTGCTCTGCGCTTCGT-3′

TGF-*β*1	Forward: 5′-GTCATAGATTGCATTGTTGC-3′
	Reverse: 5′-AAGGAGACGGAATACAGGG-3′

MMP-1	Forward: 5′-GATGGATCCCAAGCCATATATGGACGTTCC-3′
	Reverse: 5′-TTGGAATTCCGGACTTCATCTCTGTCGG-3′

NF-*κ*B	Forward: 5′-GAAGAAGCGAGACCTGGAG-3′
	Reverse: 5′-TCCGGAACACAATGGCCAC-3′

Smad3	Forward: 5′-CTGGCTACCTGAGTGAAGAT-3′
	Reverse: 5′-GTTGGGAGACTGGACGAA-3′

*β*-actin	Forward: 5′-GGAGATTACTGCCCTGGCTCCTA-3′
	Reverse: 5′-GACTCATCGTACTCCTGCTTGCTG-3′
